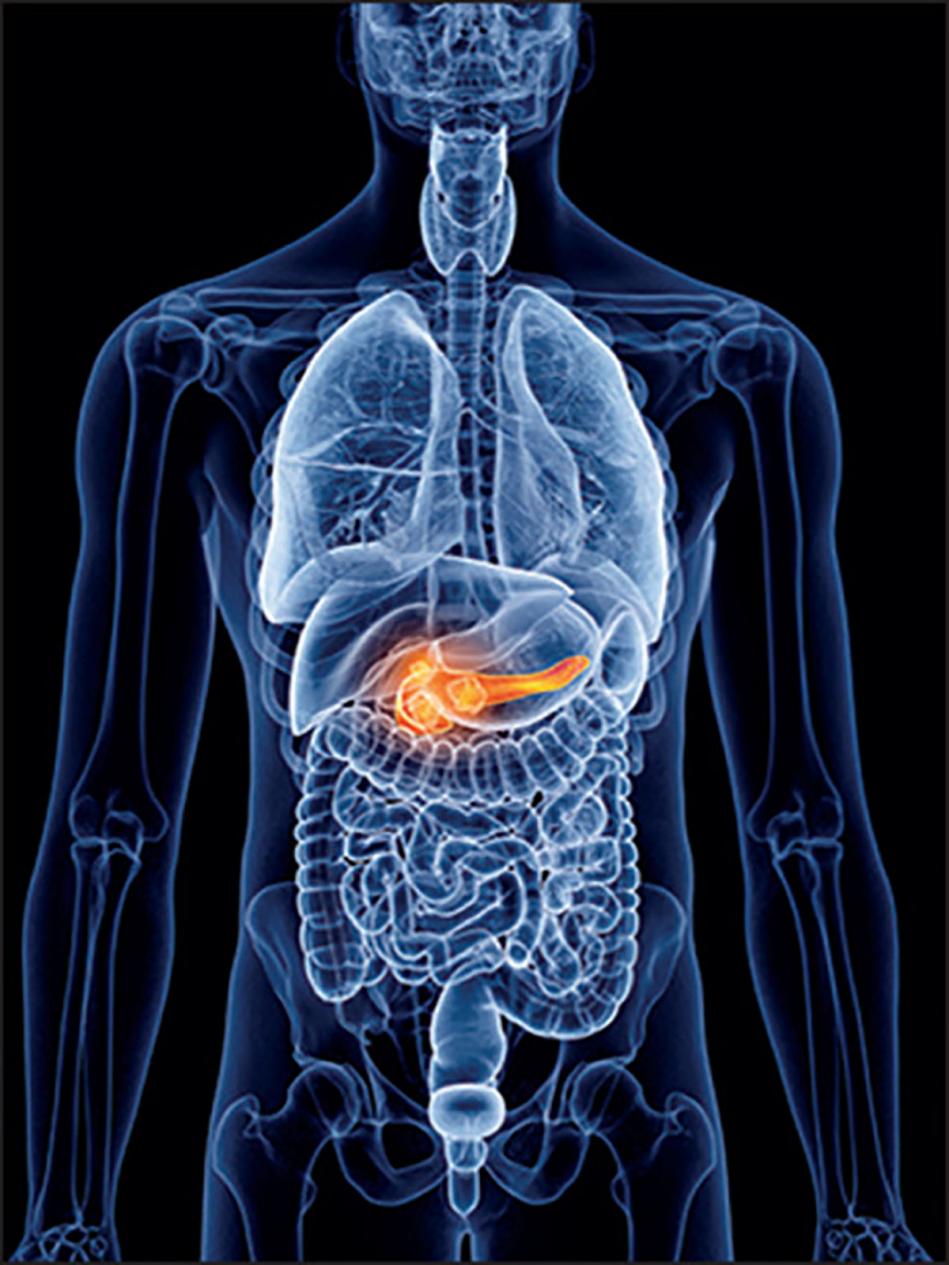# Pancreatic cancer: a growing burden

**DOI:** 10.1016/S2468-1253(19)30323-1

**Published:** 2019-10-21

**Authors:** Alison P Klein

**Affiliations:** Department of Oncology, Sidney Kimmel Comprehensive Cancer Center, and Department of Pathology, Sol Goldman Pancreatic Cancer Research Center, Johns Hopkins School of Medicine, Baltimore, MD 21231, USA

Pancreatic cancer, once considered a rare cancer, is a growing cause of cancer mortality worldwide. The global burden of pancreatic cancer is clearly shown by the GBD 2017 Pancreatic Cancer Collaborators:^1^ the worldwide annual incidence increased by 2·3 times from 1990 to 2017, from 196 000 (95% uncertainty interval 193 000–200 000) cases in 1990 to 441 000 (433 000–449 000) cases in 2017. Much of this increase is due to population ageing. Age is the strongest risk factor for pancreatic cancer, with death rates increasing throughout the lifespan from less than five per 100 000 person-years in the fourth decade of life to more than 60 per 100 000 person-years at the start of the eighth decade.^1^ Thus, as lifespan increases worldwide, the overall burden of pancreatic cancer will also increase.

In 2012, 8% of the global population was older than age 65 years.^2^ Just 3 years later, this proportion increased to 8·5%, and by 2050 it is expected to be 16·7%. The greatest increase in the proportion of the population aged older than 65 years will be in Asia, Australia, Europe, and many regions in Latin America.^2^ As shown by the GBD Collaborators,^1^ the current agestandardised death rates of pancreatic cancer are highest in western Europe, high-income North America, high-income Asia Pacific, and Southern Latin America. Thus, it must be noted that regions with the greatest projected growth in the number of individuals aged older than 65 years are the same regions with the highest rates of pancreatic cancer when adjusting for age. As such, the recent increases in the number of pancreatic cancer cases will only continue.

In addition to an ageing population, improvements in the ability to diagnose pancreatic cancer also play a part in the increasing burden of this disease. The burden of pancreatic cancer remains low in low-income settings not only because of shorter life expectancies, but also as a result of poor access to the medical care necessary to diagnose this disease. Imaging technology (CT, endoscopic ultrasound, or MRI) with expert pathological assessment of tissue is required for diagnosis. This lack of access is reflected in the lack of high-quality data about pancreatic cancer incidence and deaths for low-income nations in the GBD study.^1^ Furthermore, the observed rapid doubling of the age-adjusted risk for pancreatic cancer in some regions is likely due, in part, to regional improvements in the diagnosis and tracking of pancreatic cancer.

However, even in African nations, many of which had low pancreatic cancer rates in the GBD study,^1^ the proportion of the population aged older than 65 years is expected to almost double, to an estimated 6·7% by 2050.^2^ Thus, even without improvements in the healthcare system, increases in the incidence of pancreatic cancer in Africa are anticipated over the next few decades.

Although the large changes in population demographics and improvements in diagnosis are important drivers of the increased burden of pancreatic cancer observed over the past 25 years, changes in modifiable risk factors also play a part. Although genetic studies have shown that 20–30% of pancreatic cancer risk is due to inherited genetic factors,^3^ environmental risk factors including cigarette smoking,^4^ alcohol consumption,^4^ high body-mass index,^5^ and diabetes have important roles in pancreatic cancer risk.

Over the past 25 years, the prevalence of these risk factors has shifted both globally and nationally. In higherincome countries, particularly North America and Europe, the prevalence of cigarette smoking has decreased.^6^ However, in Asia and in many lower-income nations, rates continue to be high.^7^ By contrast, rates of obesity and diabetes continue to rise worldwide. In a recent report by WHO, the global prevalence of obesity has tripled since 1975.^8^ Individuals who are obese have a 20–50% increased risk of pancreatic cancer.^5^ Similarly, rates of alcohol intake and diabetes have risen in the past two decades and these increases are projected to continue.^9,10^

In summary, the recent increase in the global burden of pancreatic cancer^1^ will only continue given the ageing of the population and the projected rise in prevalence of many risk factors for this almost uniformly deadly cancer. Policy makers need to prepare for the continued rise in pancreatic cancer incidence. Earlier diagnosis, improved treatments, and efforts to reduce modifiable risk factors are needed to help to reduce this growing cause of cancer mortality.

## Figures and Tables

**Figure:**